# Impact of Seed Exudates on Growth and Biofilm Formation of *Bacillus amyloliquefaciens* ALB629 in Common Bean

**DOI:** 10.3389/fmicb.2017.02631

**Published:** 2018-01-09

**Authors:** Samuel J. Martins, Flávio H. V. Medeiros, Venkatachalam Lakshmanan, Harsh P. Bais

**Affiliations:** ^1^Department of Plant Pathology, Universidade Federal de Lavras, Lavras, Brazil; ^2^Department of Plant and Soil Sciences, Delaware Biotechnology Institute, University of Delaware, Newark, DE, United States

**Keywords:** seed coating, PGPR, abiotic stress, sporulation, rhizobacteria, drought, legume, plant–microbe interaction

## Abstract

We aimed to unravel the events which favor the seed-rhizobacterium *Bacillus amyloliquefaciens* strain ALB629 (hereafter ALB629) interaction and which may interfere with the rhizobacterium colonization and growth on the spermosphere of common bean. Seed exudates from common bean were tested *in vitro* for ALB629 biofilm formation and bacterial growth. Furthermore, the performance of ALB629 on plant-related variables under drought stress was checked. Seed exudates (1 and 5% v/v) increased ALB629 biofilm formation. Additionally, the colony forming units for ALB629 increased both in culture and on the bean seed surface. The bean seed exudates up-regulated biofilm operons in ALB629 *TasA* and *EpsD* by ca. two and sixfold, respectively. The high-performance liquid chromatography (HPLC)-coupled with MS showed that malic acid is present as a major organic acid component in the seed exudates. Seeds treated with ALB629 and amended with malic acid resulted in seedlings with a higher bacterial concentration, induced plant drought tolerance, and promoted plant growth. We showed that seed exudates promote growth of ALB629 and malic acid was identified as a major organic acid component in the bean seed exudates. Our results also show that supplementation of ALB629 induced drought tolerance and growth in plants. The research pertaining to the biological significance of seed exudates in plant–microbe interaction is unexplored field and our work shows the importance of seed exudates in priming both growth and tolerance against abiotic stress.

## Introduction

Abiotic stress regimes, such as drought, may interfere on plant growth and development. Of late, drought occurrences have alarmed growers around the world. The severity of drought as a major abiotic stress in growing of major staple crops is a genuine concern for global agriculture (Cook et al., 2015). On the other hand, utilization of “biologicals” or beneficial microbes has been shown to be an eco-friendly solution that can increase plant tolerance and reduce drought impact on plant development (Kavamura et al., 2013; Vurukonda et al., 2016). Although the effect of beneficial microbes on plants is discussed in the literature, there has been comparatively much less progress in elucidating the mechanisms that are involved in beneficial microbes’ effect(s) on plant performance against drought stress, with the exception of the best studied model systems (Staudinger et al., 2016).

Among the plant beneficial microbes are the plant growth-promoting bacteria (PGPB), which when applied to seeds or roots can colonize plant spermosphere or rhizosphere and then benefit plants in many ways (Lakshmanan et al., 2014; Spence et al., 2014; Martins et al., 2015; Rosier et al., 2016). Various studies have shown that the secretions in the rhizosphere can stimulate or inhibit the microbial growth (Randy et al., 2009; Hao et al., 2010; Ling et al., 2011) and may play a major role in recruitment and colonization of beneficial rhizobacteria (Rudrappa et al., 2008; Dutta and Podile, 2010; Huang et al., 2014). Recent efforts (Rudrappa et al., 2008; Chen et al., 2012) showed that root exudates of tomato and *Arabidopsis* could increase *Bacillus subtilis* biofilm formation and also that malic acid was present as a root exudate component involved in this process.

Biofilm are ubiquitous communities of tightly associated bacteria encased in an extracellular matrix of polymeric substances, such as exopolysaccharides (EPS), proteins, and sometimes DNA (Flemming et al., 2016). In *B. subtilis*, this extracellular matrix is composed mainly of EPS and the protein TasA, which polymerizes and forms amyloid-like fibers (Branda et al., 2006; Romero et al., 2010). Besides, Spo0A is a regulator DNA-binding protein for the *B. subtilis* sporulation, which also has been found to regulate biofilm formation (Lopez et al., 2009). A gain in biofilm formation may increase the potential of a beneficial bacterium to sustain the plant’s health under abiotic stresses. There are various reports regarding the role of root exudates on rhizospheric interactions (Badri et al., 2013; Chaparro et al., 2013a,b). However, much less information is available about seed exudates, whether it may or may not favor rhizobacteria colonization. Analogous to the rhizosphere, the spermosphere comprise mostly by the carbon compounds released into the soil zone, which surround the seed once the seed begins to imbibe and germinate. These compounds represent the major source of energy for the surrounding microbial communities in the spermosphere (Nelson, 2004). For commercial viability of microbial agents to be used as inoculants, it is important to understand how beneficial rhizobacteria interact with seeds, as the microbial inoculants are routinely applied as seed treatments. Seed treatment increases the time of contact between plant and the beneficial microbes, besides being a cheaper alternative to maintain plant health compared to the aerial spraying or drenching method. In this study, we aimed to unravel the events which favor the seed–rhizobacteria interaction, particularly *Bacillus amyloliquefaciens* ALB629, and which may interfere with the rhizobacterium colonization and growth on the spermosphere. ALB629 strain was obtained endophytically from healthy cacao trees (Medeiros et al., 2009). The bacterium is preserved in peptone glycerol at -80°C and deposited at Bacteriology Laboratory of Universidade Federal de Lavras (UFLA), Brazil. ALB629 has been proved to promote common bean growth as well as control bacterial wilt (*Curtobacterium flaccumfaciens* pv. *flaccumfaciens*) even under heat stress (Martins et al., 2013, 2014).

Here, we examined the importance and role of seed exudates from common bean on *B. amyloliquefaciens* ALB629 biofilm formation and hypothesized that seed exudates of common bean trigger *SpoA* and *TasA* to form biofilm its persistence in the spermosphere and improve plant’s performance against drought.

## Materials and Methods

### Seed Sterilization

*Phaseolus vulgaris* (common bean) seeds cultivar Lariat were surface sterilized by soaking in 4% sodium hypochlorite for 4 min followed by three washes with sterile water. Then, the seeds were swirled in 70% ethanol for 3 min and washed three times with sterile water.

Rice seeds cultivar M104 were surface sterilized in 4% sodium hypochlorite for 10 min followed by three washes with sterile water. Then, seeds were swirled in 70% ethanol for 10 min and extensively washed with sterile water.

### Preparation of Seed Exudates

Since we were interested in studying a possible malic acid present in the seed exudate and since malic acid is a polar molecule we collected seed exudates by soaking either common bean or rice seeds in MgCl_2_ solution (0.2%) for 2, 24, and 48 h. Six seeds per 15 ml tubes (2 g seeds l^-1^) with six replicates were used. Seed exudates were passed through 0.22 μm pore filter membranes and checked for eventual contamination by plating an aliquot of 50 μl in LB medium (10 g l^-1^ tryptone, 5 g l^-1^ yeast extract, 10 g l^-1^ NaCl). Plates were incubated at 28°C for 24 h and then checked for bacterial growth.

ALB629 was grown overnight in LB medium and washed in MgCl_2_ as described above and re-suspended to a final density of OD_600_ = 0.8 in minimal medium (MSgg) [19.52 g l^-1^ morpholinepropane sulfonic acid (MOPS) 100 mM (adjust pH to 7.0), 5 ml l^-1^ glycerol, 5 g l^-1^ glutamate, 0.41 g l^-1^ MgCl_2_, 0.87 g l^-1^ potassium phosphate (pH 7), 0.05 g l^-1^ tryptophan, 0.10 g l^-1^ phenylalanine, 0.10 g l^-1^ CaCl_2,_ 0.013 g l^-1^ FeCl_3_, 100 μl MnCl_2_ (from stock of 50 mM), 100 μl thiamine (from stock of 2 mM), and 100 μl ZnCl_2_ (from stock of 1 mM)] (Branda et al., 2001).

The obtained seed exudates were tested for its implication on the rhizobacteria biofilm formation, three concentrations of seed exudates [0.4, 1, and 5% v/v, or MgCl_2_ solution (control)] were mixed with the bacterial suspension using the method of O’Toole and Kolter (1998). Samples of 100 μl of the diluted cells were aliquoted into sterile 96-well microtiter plates with eight wells per treatment as replicates. Plates were covered and incubated at 30°C without agitation for 48 h. Cells that had adhered to the well walls were treated with 0.1% crystal violet for 10–15 min at 25°C without agitation; the plates were drained of liquid via pipet, gently rinsed several times with water, and allowed to dry at room temperature. The dye that had stained the cells was solubilized in 200 μl of 95% (v/v) ethanol. Biofilm formation was quantified by measuring the optical density at 630 nm for each well using Wallac 1420 Manager plate reader.

### ALB629 Growth in the Presence of Seed Exudates

To check the ALB629 growth with bean seed exudate presence, the bacteria were grown as described previously and re-suspended in MSgg to get a final density of OD_600_ = 0.2. Then, bacterial suspensions were grown for 4 and 10 h at 28°C, 220 rpm with seed exudates at 1%. As a control, the bacteria were grown without seed exudates. At the referred time points, ALB629 suspensions were diluted and 10 μl was poured on Petri dishes with LB media. Plates were incubated at 28°C for 48 h and cells were counted for the number of colony forming units (CFU). Six replicates (*n* = 6) were used, three of which were biological replicates and two of which were technical replicates for treatment.

### Effect of Seed Exudates on Seed Treatment with ALB629 Seen under Confocal Microscope

ALB629 was grown overnight in LB medium, washed in MgCl_2_ as described above, and re-suspended in MgCl_2_ solution to a final density of OD_600_ = 0.8. Common bean seeds were treated with the bacterial solution for 2 h. In one of the treatments 1% of bean seed exudate, which was taken from 24 h of incubation, was added to the final bacterial solution. As a control, seeds were treated with water. Seeds were then fixed in 2% glutaraldehyde for 2 h, washed in filtered PBS buffer, and finally stained in SYTO^®^13 Green Fluorescent Nucleic Acid Stain for 7 min. The seeds were analyzed in LSM 710 confocal microscopy.

### RT-PCR and Semi-quantification

For this experiment, 1% of seed exudate concentration from the 24 h seed exudate was used to check the expression of the following *TasA* (biofilm), *EpsD* (EPS), and *Spo0A* (sporulation) at 0, 4, and 10 h of bacterial growth. However, prior to gene expression, normalization was performed with the housekeeping gene *RecA*, which has been used as a reference gene in various studies involving bacteria (Rivas et al., 2005; McMillan and Pereg, 2014; Da Silva et al., 2016). The bacterium was grown overnight in LB medium and washed in MgCl_2_ as described above and re-suspended in MSgg medium to a final density of OD_600_ = 0.2A. Bean seed exudate at 1% was added into the bacterial suspension and grown for 0, 4, or 10 h at 28°C 220 rpm. As a control, the bacteria were grown without seed exudates. Total RNA was isolated using the Macherey-Nagel^TM^ Kit from 1.5 ml bacterial suspension, following the manufacturer’s instructions. For 0 h, RNA extraction was performed from the bacterial suspension samples at the first time point of bacterial growth. For RT-PCR, cDNA was synthesized using M-MuLV reverse transcriptase (New England Biolabs) from 500 ng of RNA according to the Applied Biosystems protocol, followed by PCR amplification using DyNAzyme II DNA polymerase (DyNAzyme). The gene-specific primers for the genes *RecA, TasA, EpsD*, and *Spo0A* are listed in **Table 1** (Qiu et al., 2014). Three experimental replicates for each treatment were used in two different experiments. The band intensity was taken from agarose gel images and quantified by Image J 1.47v^1^ (National Institute of Health, United States).

**Table 1:** Gene-specific primers and annealing temperatures used for RT–PCR.

Primer	Primer sequence (5′–3′)	Annealing temperature (°C)	Reference
*RecA* forward	AAAAAACAAAGTCGCTCCTCCG	55	Qiu et al., 2014
*RecA* reverse	CGATATCCAGTTCAGTTCCAA		
*TasA* forward	GGATTTCCTCAGCCAGTTTG	55	This study Gene ID: FJ713580.1
*TasA* reverse	TTTCGGAACTCCGTCGTACT		
*EpsD* forward	TTTTCGGCAGCCATTCCTTC	55	Qiu et al., 2014
*EpsD* reverse	TGTATCTGACATTGTGCGGTTT		
*Spo0A* forward	GACGGACTTGCGGTTTTAGA	32	This study Gene ID: AY672772.1
*Spo0A* reverse	GCCGATTTCATGGATAATGC		

### Identification Analysis of Malic Acid in the Seed Exudates

Identification of malic acid in the exudates from non-inoculated seeds was carried out using high-performance liquid chromatography (HPLC)-MS Q Exactive, Thermo Scientific, H-ESI. The device was operating in a full negative mode scan at a resolution setting of 70,000, with a spray voltage of spray 4000 V, sheath gas flow rate set to 12 arb. units with three auxiliary arb. units, capillary temperature of 300°C, and S-lens of 60 arb. units. The sample at 600 ppm was diluted in methanol with 0.1% formic acid. The standard solution used was prepared by malic acid Synth PA and Milli-Q water. Data were obtained by Xcalibur software.

### Selection of the ALB629^rif-nal^

To study the ALB629 colonization of 22-day-old bean seedlings submitted to drought stress, a spontaneous rifampicin/nalidixic acid (ALB629^rif-nal^) was selected from a *B. amyloliquefaciens* strain ALB629 based on Medeiros et al. (2009). To select the ALB629 with the selection marker, increasing amendments of both antibiotics were added to the LB medium up to 100 ppm at each bacterial culture. The spontaneous mutant strain was previously compared to the wild type for its growth and biological control efficacy and the mutation did not impact such variables (Martins et al., 2014).

### *In Vivo* Drought Stress Experiment

In order to find out the capability of ALB629 in sustaining plant growth under abiotic stress, we selected the best time point regarding seed treatment in the previous assay to proceed with the seed treatment. A treatment using malic acid (0.5%) (Kleijn et al., 2010) was used in order to find out its effect on biological seed treatment. For both treatments, pH was brought to 5.6–5.8. Bacterial suspensions were submitted to the seed treatment for 2 h from a bacterial culture of 2-day-old as described before. As a control water and water + malic acid was used in the drought assays.

Pots filled with 0.3 l sterilized ultralite top soil and with one seedling per pot were kept under growth chamber conditions: 200 μE m^-2^ s^-1^ 12 h of photoperiod of 23°C/15°C (day/night), and relative humidity of 55/70% (day/night) according to Shevyakova et al. (2013) for abiotic stress.

Seedling emergence was recorded daily and used to calculate the speed emergence index (SEI) as described by Teixeira and Machado (2003). When the first fully expanded leaves appeared, irrigation was suspended for 2 weeks, the time point in which plants started wilting. Then, plants were collected and checked growth promotion parameters: fresh and dry weights, fw/dw ratio, stem size, measurements of leaf width and length. Additionally, root tissue was washed twice with SDW, weighed, and transferred to 2 ml tubes containing 900 μl of SDW. The tissue was crushed, vortexed, and serially diluted to 10^-5^ and then plated by spreading on LB medium with 100 ppm of rifampicin/nalidixic acid. Plates were incubated at 28°C for 48 h and data were transformed to log_10_ CFU g of fresh tissue weight^-1^.

### Experimental Design and Statistical Analysis

All experiments were carried out randomized complete block with 8 and 10 replicates, respectively, for the *in vitro* and *in vivo* (drought stress) tests. Data were submitted to one-way analysis of variance (ANOVA) and for significant means Scott–Knott test, Tukey’s multiple range tests, or Student’s *t*-test (*p* < 0.05) were applied. For all analyses, the assumptions of normality of variance were checked by the Shapiro–Wilk test and no transformation was necessary.

## Results

### Biofilm Formation in ALB629 Amended with Seed Exudate

**FIGURE 1:**
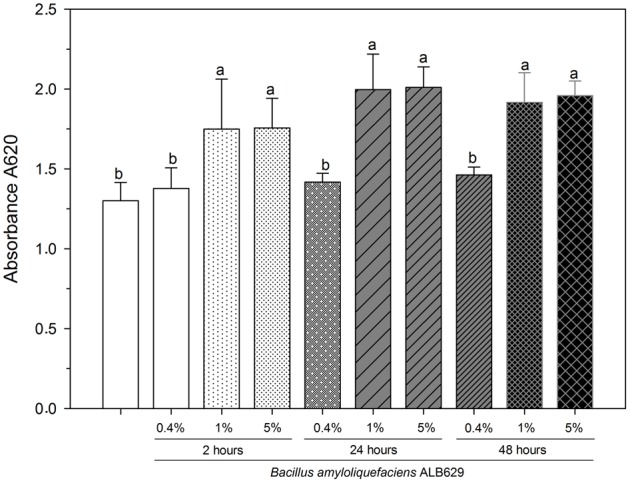
*In vitro* biofilm formation by *Bacillus amyloliquefaciens* ALB629 in 96-well plates with different concentrations of bean seed exudates from different time points (2, 24, and 48 h). The white bar represents the negative control (ALB629 without seed exudate treatment). Bars with the same letter are similar at the 5% level according to Scott–Knott’s test. The line on each bar represents ±SE.

The effects of seed exudates of common bean on ALB629 growth and biofilm formation were tested. Different concentrations (0.4, 1, and 5% v v^-1^) of seed exudates collected were directly tested on biofilm formation in ALB629. Seed exudates were incubated and streaked on LB plates to negate contamination. Common bean seed exudates at the concentrations of 1 and 5% increased biofilm formation by ALB629 regardless of the time point (2, 24, and 48 h) that they were collected when compared to the negative control (ALB629 without seed exudate) (**Figure 1**). To evaluate the specificity of the seed exudation to trigger biofilm formation in ALB629, we checked the seed exudates from a non-legume plant (rice) on biofilm formation in ALB639. Our data show that rice seed exudate also triggers biofilm formation in ALB629 similar to bean seed exudates, suggesting the non-specificity of seed exudates to induce biofilm formation (Supplementary Figure 1).

### Growth and Biofilm Formation of ALB629 in the Presence of Bean Seed Exudate

**FIGURE 2:**
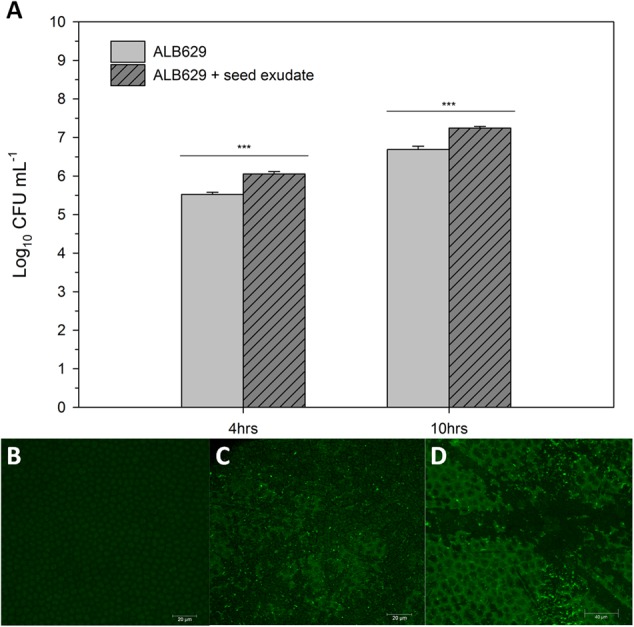
**(A)** Seed exudates from common bean stimulate ALB629 growth when the LB liquid medium was amended with (1%) (v/v) of the seed exudate and growth checked at 4 and 10 h. ^∗∗∗^Significant at the 0.001 probability level by Student’s *t*-test. The line on each bar represents ±SE; **(B)** Surface of common bean seeds under confocal microscope for seed treated with water (control), **(C)** seed treated with ALB629 only and, **(D)** seed treated with ALB629 + seed exudate (1%) (v/v). The fluorescent punctate spots show an abundance of ALB629 on common seed coats. Each image is represented by a z stack of 115 images.

To evaluate the biofilm, the bacterial colony formation units (CFU) in ALB629 exposed to seed exudates were also evaluated. There was a higher growth for ALB629 in the presence of common bean seed exudates for post 4 (*p* = 0.0001) and 10 h (*p =* 0.0002) of growth (**Figure 2A**). To validate the biofilm formation and bacterial association triggered by seed exudates, confocal microscopy was utilized. Confocal micrographs showed that addition of 1% seed exudates triggered bacterial association on seed surface (**Figures 2B–D**).

### Biofilm Operons Gene Expression in ALB629 in the Presence of Bean Seed Exudate

**FIGURE 3:**
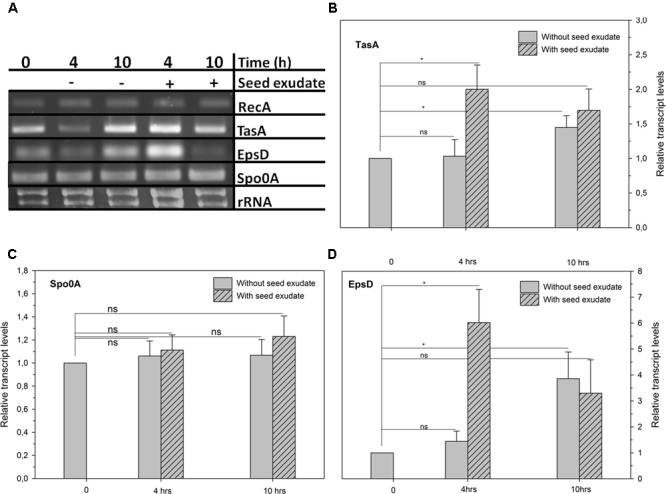
**(A)** Relative expression of biofilm-related genes: biofilm (*TasA*, **B**), sporulation (*Spo0A*, **C**), and exopolysaccharide-related genes (*EpsD*, **D**) of *B. amyloliquefaciens* ALB629 cultivated in nutrient both amended or not with the seed exudate at 1% (v/v) of 24 h exposure incubation of seeds at room temperature. *RecA* = housekeeping gene. ^∗^Significant difference at the 0.05 probability level by Student’s *t*-test. ns = not significant. The line on each bar represents ±SE.

Seed exudates from common bean up-regulated biofilm operons in ALB629 in a *TasA*-and *EpsD*-dependent manner at 4 h by about ∼two and sixfold, respectively, higher than the untreated control (**Figures 3A,B,D**). On the other hand, in the absence of added seed exudate, ALB629 biofilm operons *TasA* and *EpsD* increased their expressions only at 10 h by about 1.5- and 4-fold, respectively. However, the difference between the treatments regarding *Spo0A* was not statistically significant regardless of the time point tested (**Figure 3C**).

### Biochemical Analysis of Bean Seed Exudate

**FIGURE 4:**
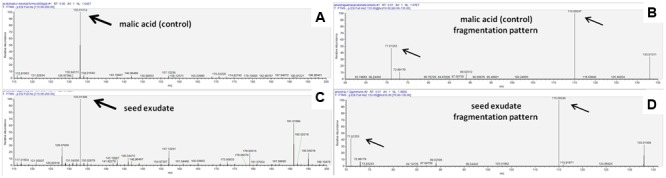
**(A)** HPLC-MS spectra of malic acid p.a. (pro-analysis); **(B)** malic acid p.a. fragmentation pattern; **(C)** spectra of exudate from non-inoculated seeds cultivar Lariat; **(D)** fragmentation pattern of the exudate.

When the seed exudates were analyzed by HPLC-MS a molecule was found at the relative abundance of 133.01308 (**Figure 4C**) and with a fragmentation pattern of 115.00246 and 71.01253 (**Figure 4D**), which showed the same relative abundance as malic acid PA 133.01314 (**Figure 4A**) and its fragmentation pattern 115.00247 and 71.01253 (**Figure 4B**), confirming the presence of malic acid as a major organic acid in the seed exudates of common bean.

### Role of ALB629 and Malic Acid on Drought Tolerance in Common Bean

**FIGURE 5:**
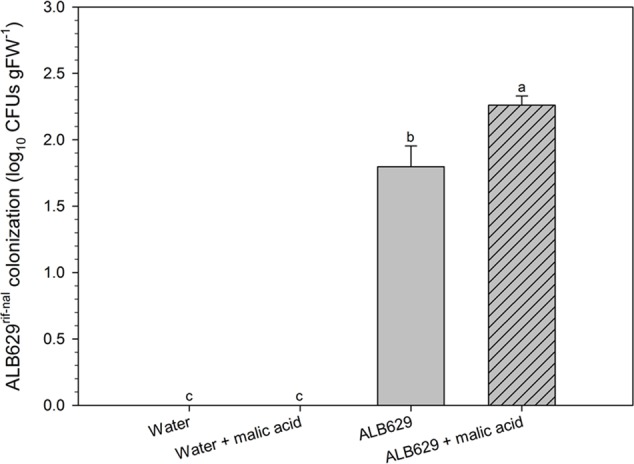
Effect of seed treatment of ALB629^rif-nal^ with malic acid on the bacterial population on the roots. Bars with the same letter are similar at the 5% level according to Tukey’s multiple range test. The line on each bar represents SE. Plates at the bottom show the bacterial dilutions (10^-1^, 10^-2^, 10^-3^, 10^-4^) for each of the four treatments.

When subjected to drought trials for 2 weeks, seedlings from seeds treated with ALB629^rif-nal^ and malic acid showed higher colonization by the rhizobacteria compared to the lone ALB29 treatments (**Figure 5**).

**FIGURE 6:**
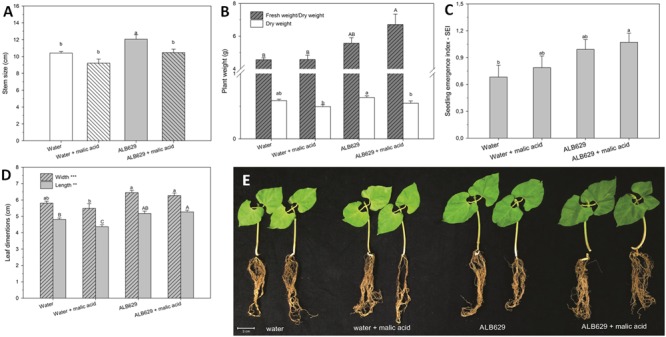
Effect of seed soaking of ALB629 or water amended or not with malic acid at the seed treatment on **(A)** stem size; **(B)** biomass accumulation; **(C)** seedling emergence index; **(D)** leaf dimensions; **(E)** bean seedlings after 2 weeks under drought stress. Bars with the same letter are similar at the 5% level according to Tukey’s multiple range test. The line on each bar represents SE.

In addition, the seed treatment with ALB629 and malic acid increased the seedling emergence index (**Figure 6C**), leaf length (**Figure 6D**), plant fresh weight, and plant water holding capacity compared to the water control or water associated with malic acid (**Figures 5B, 6B,E**). Water holding capacity was represented as a ratio between fresh weight and dry weight. Moreover, ALB629 itself could increase the seedling stem size (**Figure 6A**). When the time point used for the biological seed treatment was tested, the optimal result regarding the number of bacterial cells recovered from the seeds was 2 h of seed immersion using a 2-day-old bacterial suspension (Supplementary Figures 2a,b).

## Discussion

The field of functional microbiome has seen a dramatic upsurge in finding microbial strains that may act as both biocontrol and growth promotional agents for plants (Lakshmanan et al., 2015). Of late, different lines of research has shown that single microbial isolates impart various plant growth promoting traits, yet very few studies show the mechanism by which microbial strains colonize or protect plants. The research pertaining to carbon turn over in the rhizosphere has led to an understanding the root exudates may trigger microbial activity and colonization in soil (Bais et al., 2006). There is very limited knowledge that entails secretions from other plant organs that may facilitate microbial activity belowground. In here, we show that *B. amyloliquefaciens* ALB629 is capable of forming biofilm and that seed exudate from common bean may accelerate the bacterial growth and boost the biofilm formation in ALB629. Although the increase in the bacterial growth when the seed exudate was amended was less than 1 log_10_ (**Figure 2A**), we have observed a much higher biofilm formation on the seed surface, therefore, such amendment seem to act as a trigger of the biofilm formation gene cassette, which in turn assure the bacterial attachment to the seed and the biological activity of the symbiont to the plant.

Malic acid was recognized as a component in seed exudates of common bean that triggered both biofilm formation and increased the bacterial seed colonization and binding protects the plants against abiotic stress (drought). Therefore, our finding foster a seed treatment technology approach that could be adopted by the industry to increase the bacterial establishment in the spermosphere and the plant protection benefit exerted by the symbiont.

As seeds imbibe water and germinate, they passively release exudates, forming a chemical gradient around seeds (Schiltz et al., 2015). The spermosphere is a primary colonization area for different kinds of microorganisms, including pathogens and beneficial microorganisms, such as plant growth promoting rhizobacteria. Among the many advantages of beneficial microorganisms, these organisms can protect the seeds against soil-borne pathogens by creating a biological shield (Chen and Nelson, 2008) and can trigger the plant defense through priming (Pozo et al., 2008; Conrath et al., 2015). These benefits represent an ecological advantage factor against not only biotic competitors but also abiotic stresses, by which plants are constantly challenged. The results showed that when ALB629 was grown in the presence of seed exudate there was a stronger and faster upregulation of biofilm-related operons (*TasA* and *EpsD*) compared to the untreated control. In the presence of seed exudate both *TasA* and *EpsD* showed a faster upregulation (at 4 h); however, its expression decreased at 10 h. On the other hand, when the exudate was not present the *TasA* and *EpsD* expression only increased at 10 h.

It is known that biofilm is composed mainly of an EPS and the amyloid-like protein TasA, encoded, respectively, by *EpsD* and *TasA* operons (Chen et al., 2012; Romero et al., 2014).

By HPLC-MS analysis, we identified malic acid as a component of seed exudate, which fragmented in with the similar pattern as shown previously (Mena et al., 2012). In our drought stress test, seedlings from seeds treated with ALB629 and with an exogenous malic acid supplementation showed a promotion in growth with the increased plant water holding capacity. Under *in vitro* conditions, Zhang et al. (2010) described a similar drought tolerance induction in *Arabidopsis* by the supplementation of *B. subtilis* GB03 by modulating osmotic pressure in the medium. Modulation of osmotic pressure by supplementing chemicals such as mannitol may induce drought-like symptoms (Zhang et al., 2010). Additionally, a higher ALB629 population was found on MA-treated plants, suggesting that malic acid was benefiting the interaction between the plant and the beneficial bacteria. In accordance with these findings, Rudrappa et al. (2008) have shown that the malic acid secreted from *Arabidopsis thaliana* roots could recruit *B. subtilis* FB17, a beneficial bacterium from soil. The malic acid has been shown to be a major tomato root exudate component involved in the process of increasing *B. subtilis* biofilm exudates (Chen et al., 2012). In addition to recruiting *Bacillus* spp., malic acid has been found to have positive chemotactic in several other microorganisms, such as *Pseudomonas fluorescens* (de Weert et al., 2002; Sood, 2003), *Ralstonia solanacearum* (Wu et al., 2015), as well as in *Glomus fasciculatum* (Sood, 2003). Comparatively to the heterotrophic bacteria that in general prefer just one carbon source (glucose) (Gorke and Stulke, 2008; Singh et al., 2008), Gram-positive bacteria, such as *Bacillus* spp. can use multiple sources of carbon (Kleijn et al., 2010; Meyer and Stulke, 2013). In contrast to glucose, the malate, which is the ionized form of MA, is commonly available in the soil and on plant surfaces (Bais et al., 2006; Rudrappa et al., 2008).

## Conclusion

The present study revealed that malic acid, an organic acid component of seed/root exudates, can be used by ALB629 to increase its biofilm formation and to reinforce its population on common bean seeds as well as in plants. The increased colonization by ALB629 induced drought tolerance and plant growth promotion in common bean. This study showed that the seed exudates from cultivated crops may favor beneficial microorganisms. The implications of our work may increase the knowledge pertaining to the characterization of seed exudates to promote microbial inoculum technology. Our long-term goal is to use this information to develop tools for predicting the efficacy of PGPB in increasing plant growth and abiotic/biotic stress tolerance.

## Author Contributions

SM was the primary person in charge of performing the assays, analyzing the data, writing, and organizing the journal submission. FM contributed by organizing the exchange program, which allowed SM to work in the United States in HB’s Lab, working with the bacterial strain ALB629 permit to be sent and used in the United States, guidance of Samuel during his Ph.D. and contributed to the manuscript writing/revision, and organizing the journal submission. VL contributed to the gene expression experiments and data analysis. HB hosted SM in his lab, worked with the bacterial strain ALB629 permit to be sent and used in the United States, and contributed to all the experiment procedures as well as manuscript writing/revision.

## Conflict of Interest Statement

The authors declare that the research was conducted in the absence of any commercial or financial relationships that could be construed as a potential conflict of interest.

## Footnotes

1 http://imagej.nih.gov/ij/
