# Supplementary material for: Impact of Seed Exudates on Growth and Biofilm Formation of Bacillus amyloliquefaciens ALB629 in Common Bean
Source: Front Microbiol. 2018 Jan 9;8:2631. doi: 10.3389/fmicb.2017.02631 (PMC5767182; doi:10.3389/fmicb.2017.02631)
Supplement: Supplementary file 1 [file Data_Sheet_1.docx]

**Supplementary figures**

**
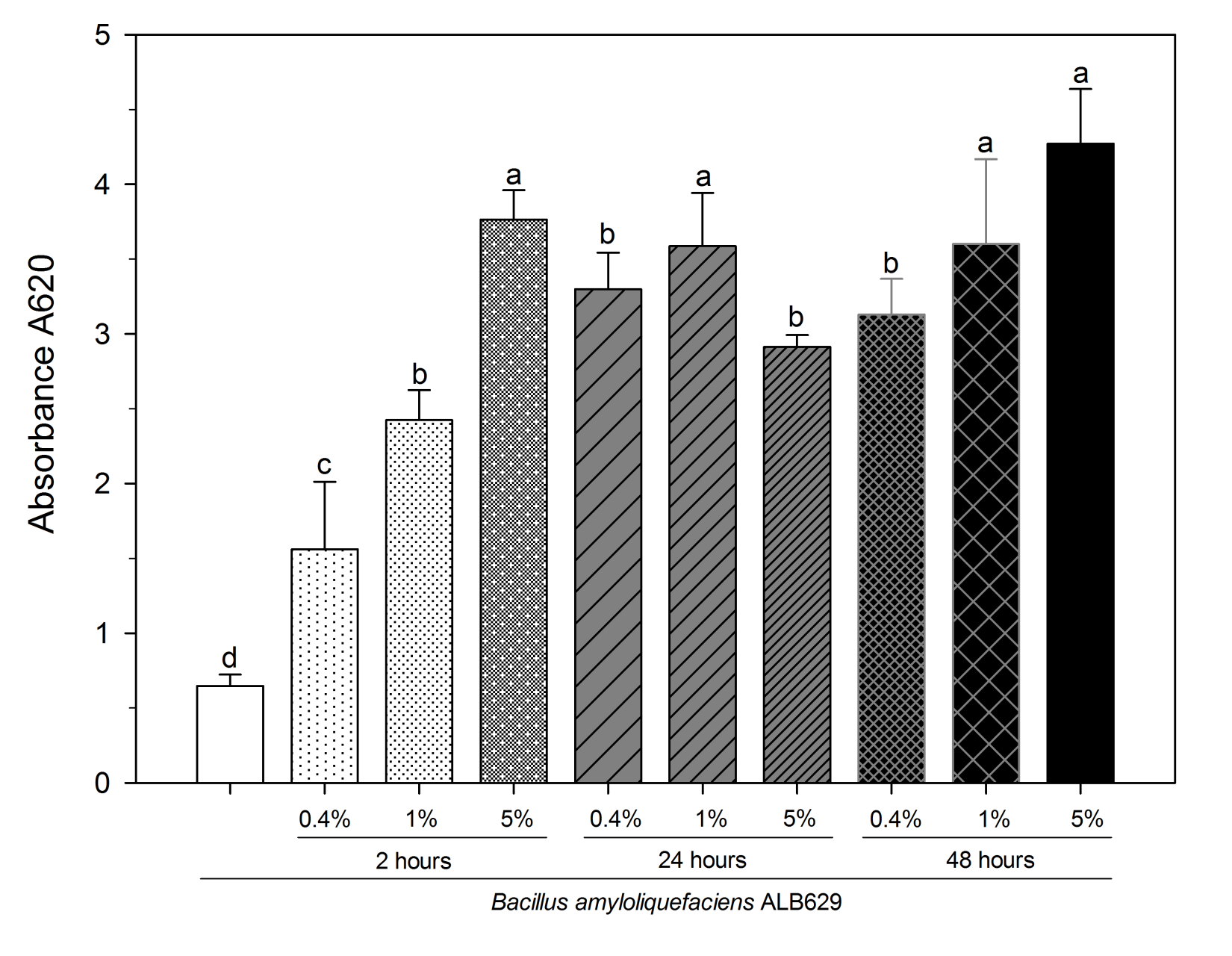
**

**Supplementary figure 1** *In vitro* biofilm formation by *B.* *amyloliquefaciens* ALB629 in 96 well plates with different concentrations of rice seed exudates from different time points (2h, 24h, and 48h). The blank bar represents the control (ALB629 without seed exudate treatment). Bars with the same letter are similar at the 5% level according to the Scott-Knott test. The line on each bar represents ±SE


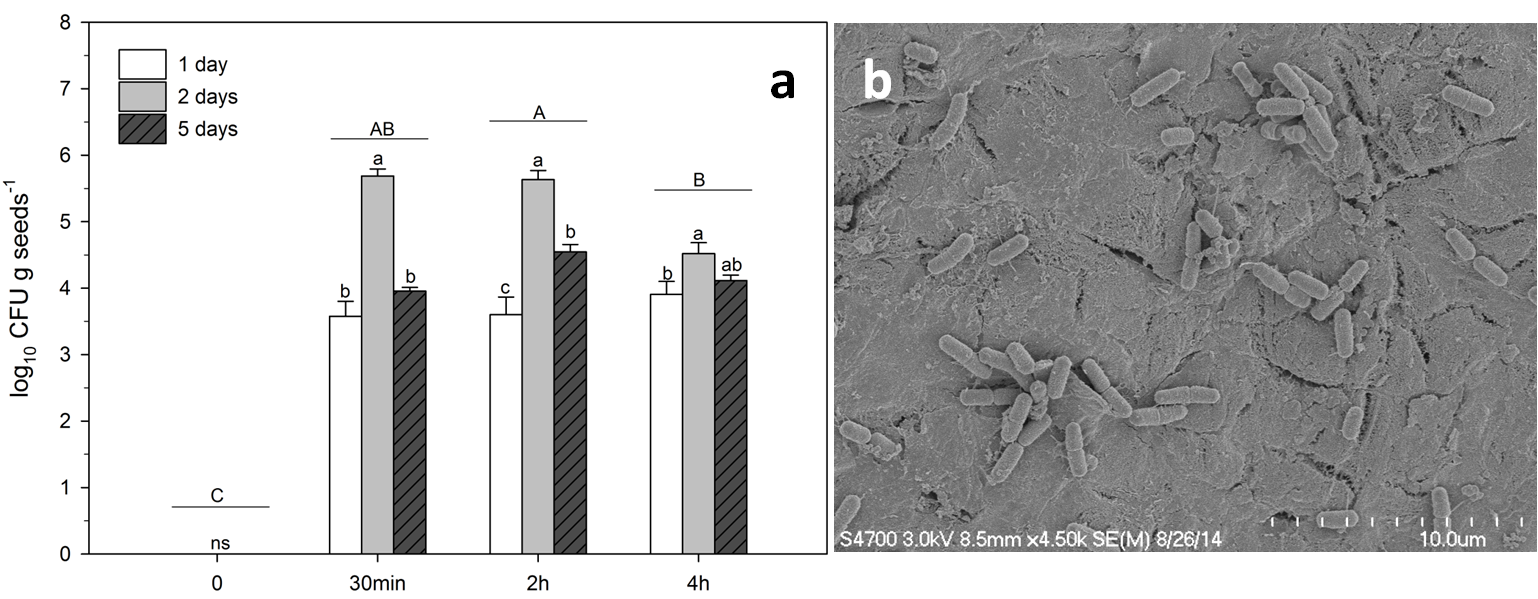


**Supplementary figure 2** (A) Time points and bacterial culture age for biological seed treatment with *B.* *amyloliquefaciens* ALB629 expressed as the number of [colony-forming unit](http://en.wikipedia.org/wiki/Colony-forming_unit)s (CFU) per g of seed (log_10_ CFU g^-1^). Bars with the same letter are similar at the 5% level according to the Scott-Knott’s test. The line on each bar represents ±SE. (B) Common bean seed colonization by *B.* *amyloliquefaciens* ALB629 from seed treatment of two hours of seed immersion in a two-day-old bacterial suspension observed through a [scanning electron microscope](http://en.wikipedia.org/wiki/Scanning_electron_microscope) (SEM)
